# Digital oncology frameworks in Africa: a scoping review of architectural patterns, digital maturity, and data equity implications

**DOI:** 10.3389/fpubh.2026.1838736

**Published:** 2026-05-28

**Authors:** Wasswa William, Andrew Ware

**Affiliations:** 1 Department of Biomedical Sciences and Engineering, Mbarara University of Science and Technology, Mbarara, Uganda; 2 Faculty of Computing, Engineering and Science, University of South Wales, Pontypridd, United Kingdom

**Keywords:** cancer registries, digital oncology, mHealth, precision oncology, system architecture, tele-oncology

## Abstract

**Background:**

Cancer mortality-to-incidence ratios in Africa remain significantly higher than in high-income settings, driven by late diagnosis, limited specialist capacity, limited access to information and fragmented surveillance systems. Digital oncology frameworks are increasingly recognised as critical enablers of cancer control and management; however, their architectural characteristics have not been systematically synthesised to inform scalable platform development and deployment. This paper systematically maps digital oncology frameworks across Africa, characterises their dominant architectural patterns, digital maturity and AI integration levels, and derives evidence-informed design recommendations for future platforms with explicit attention to how architectural choices shape health data equity across diverse African health system contexts.

**Methods:**

A scoping review was conducted following Arksey and O’Malley and Joanna Briggs Institute guidelines, with PRISMA-ScR reporting. Searches were performed across PubMed, ScienceDirect, Web of Science, IEEE Xplore, and African Journals Online. Frameworks were classified into six categories: population-based cancer registries; hospital-based oncology information systems; tele-oncology platforms; mHealth frameworks; cancer information hubs; and genomic/precision oncology systems. Data extracted included architecture type, data flow, interoperability, digital maturity, and AI integration.

**Results and discussion:**

Fifty-three frameworks were identified. Registries were predominantly centralised at Digital Maturity Level 2, with higher maturity achieved through national health system integration. Hospital oncology information systems revealed a trade-off between vendor-integrated high-performance platforms and more interoperable open-source alternatives. Tele-oncology adopted scalable hub-and-spoke architectures supporting specialist reach to underserved facilities. mHealth frameworks were largely unidirectional SMS systems, effective for community engagement but limited in clinical integration. Cancer information hubs ranged from centralised analytical repositories to DHIS2-based interoperable systems. Genomic frameworks operated as federated research networks with limited clinical translation. AI integration was limited across all categories, reflecting underlying data standardisation and architectural deficits. Critically, the structural fragmentation, interoperability gaps, and geographic concentration of higher-maturity systems in well-resourced facilities collectively constitute a health data equity deficit; systematically excluding lower-resourced populations and settings from the benefits of digitally enabled cancer care.

**Conclusion:**

Digital oncology systems in Africa are architecturally diverse but structurally fragmented. Advancing equitable cancer care requires interoperability-first, nationally embedded, and AI-ready digital architectures capable of supporting scalable, longitudinal, and inclusive oncology data ecosystems across diverse African health system contexts.

## 1 Introduction

### 1.1 Global cancer burden and the urgent imperative for digitisation

Cancer remains one of the leading causes of morbidity and mortality globally, and it currently represents a major and escalating public health challenge (1). Recent estimates from the International Agency for Research on Cancer (IARC) indicate that global cancer incidence has surpassed 20 million new cases annually, with projections suggesting continued growth driven by population ageing, demographic expansion, and epidemiological transition (2). The distribution of the cancer burden is increasingly shifting towards low- and middle-income countries (LMICs), where health systems often lack the infrastructure, workforce capacity, budget constraints, and surveillance systems required for comprehensive cancer control. In Africa, cancer incidence and mortality are rising steadily due to urbanisation, lifestyle changes, environmental exposures, and the persistent burden of infection-related malignancies such as cervical and liver cancers (3). Mortality-to-incidence ratios in many African countries remain markedly higher than in high-income regions, reflecting delayed diagnosis, inadequate screening coverage, limited specialist density, fragmented referral systems, and underdeveloped cancer registries. Within this context, digitisation is not merely an efficiency-enhancing intervention but a structural necessity for effective cancer control (4). Digital oncology infrastructures enable earlier detection through AI-assisted imaging and mobile-enabled triage systems; improve continuity of care via longitudinal electronic health records that reduce loss to follow-up; support evidence-informed resource allocation through robust population-based cancer registry data; enhance adherence to complex treatment protocols via clinical decision-support tools; facilitate data harmonisation for regional research collaboration and policy planning; and expand equitable access through tele-oncology and distributed care delivery models (4).

Africa presents a uniquely important context for digital oncology due to its large geographic expanse, highly dispersed rural populations, uneven distribution of specialist oncology services, and substantial variability in digital and healthcare infrastructure across and within countries (5). In many African settings, patients must travel long distances to access cancer diagnosis and treatment, while specialist centres are concentrated in major urban areas. These realities significantly influence digital oncology system design, favouring architectures that support decentralised service delivery, remote consultation, mobile-enabled communication, cloud-based coordination, and scalable interoperability across geographically distributed facilities. Consequently, architectural considerations such as offline functionality, low-bandwidth optimisation, hub-and-spoke communication models, and mobile-first deployment approaches become particularly relevant within African oncology ecosystems (6).

### 1.2 Digital oncology in developed health systems

High-income countries have increasingly operationalised integrated digital oncology ecosystems characterised by system-wide technological interoperability, Clinical Decision support and longitudinal data integration (7). Comprehensive oncology information systems (OIS) are embedded within hospital electronic health record (EHR) architectures, enabling coordinated management of diagnostics, chemotherapy ordering, radiotherapy planning, pharmacy workflows, adverse event monitoring, and multidisciplinary documentation in cancer units (8). National and regional population-based cancer registries provide structured, often near real-time reporting that supports surveillance, quality benchmarking, epidemiological modelling, policy support and clinical research. Furthermore, AI-enabled radiology and digital pathology platforms are being integrated into diagnostic workflows, facilitating automated lesion detection, risk stratification, and treatment response assessment (9). On the other hand, genomic sequencing platforms and molecular tumour boards increasingly inform precision oncology decision-making (10), while cloud-based collaboration systems support distributed multidisciplinary consultations across institutions. Predictive analytics tools (11) are deployed for toxicity monitoring, hospital readmission risk prediction, survivorship planning, and population-level outcome modelling. These ecosystems operate within mature national digital health strategies, supported by robust data governance frameworks, privacy regulations, and interoperability standards.

### 1.3 The African digital oncology landscape

Across Africa, digital oncology is emerging but remains heterogeneous in scope, maturity, and integration (12). Progress is evident in the development and strengthening of population-based cancer registries, which promotes standardised data collection methodologies and registry capacity building (13). Some tertiary institutions have implemented hospital-level oncology information systems to manage chemotherapy workflows, radiotherapy scheduling, and diagnostic integration (14). Mobile health (mHealth) platforms are increasingly deployed to support cervical cancer screening, follow-up, patient navigation, and adherence monitoring (15). Tele-oncology platforms mitigate workforce shortages by enabling remote consultations and virtual tumour boards, while pilot AI applications in radiology and pathology are emerging within academic centres (14). However, these initiatives frequently operate in silos, with limited interoperability between registries, hospital systems, and diagnostic platforms. AI validation datasets are often small, geographically constrained, and insufficiently representative, raising concerns regarding model generalizability and bias. Sustainability models commonly depend on donor or project-based funding, and oncology-specific digital architectures are not consistently embedded within national digital health strategies.

In addition, the implementation of digital oncology systems across Africa faces several persistent structural and operational challenges. Many health systems continue to experience unreliable electricity supply, limited broadband connectivity, inadequate cloud and data storage infrastructure, and constrained access to advanced diagnostic hardware, particularly in rural and peripheral settings (12). High procurement and maintenance costs for oncology information systems, imaging platforms, and genomic technologies further limit the scalability and long-term sustainability of digital oncology tools. Workforce shortages remain substantial, including limited numbers of oncologists, health informaticians, data scientists, biomedical engineers, and digitally trained health personnel capable of implementing and maintaining complex digital infrastructures. Interoperability between registries, hospital information systems, laboratory platforms, and national health information systems is frequently weak or absent, resulting in fragmented data ecosystems and duplicated workflows. Furthermore, governance and regulatory frameworks for data protection, cross-border data exchange, cybersecurity, AI validation, and digital health standardisation remain unevenly developed across many African countries, creating additional barriers to large-scale integration and sustainable deployment of digital oncology architectures (16). Despite these structural constraints, Africa presents distinctive opportunities. For example, high mobile phone penetration enables scalable digital interventions; cloud-native systems allow leapfrogging beyond legacy infrastructure; AI research capacity is expanding within universities; and continental policy attention towards digital transformation and non-communicable diseases is increasing.

### 1.4 Conceptualising digital oncology frameworks

**Figure 1:**
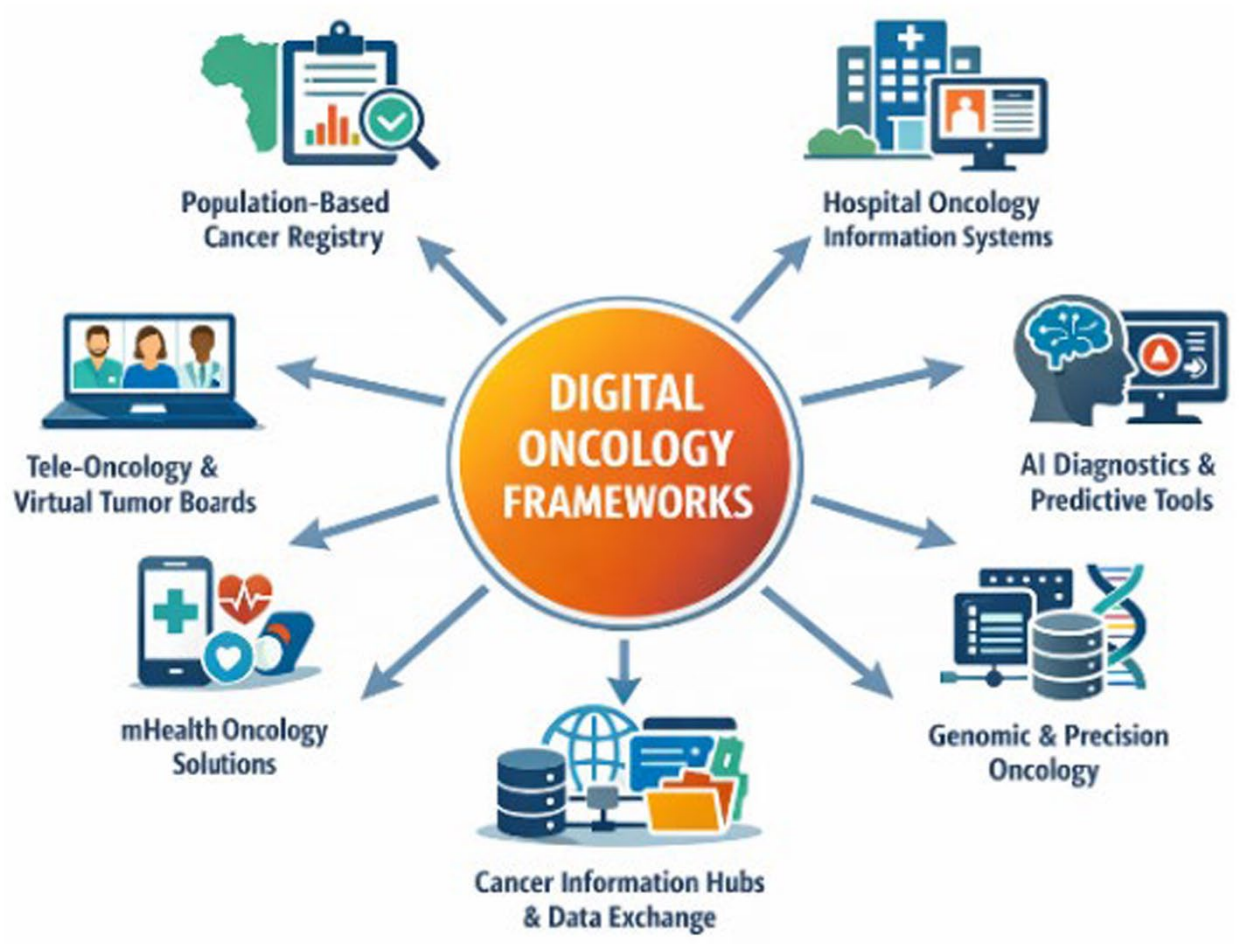
Digital oncology frameworks.

To move beyond isolated tools and pilot interventions, digital oncology must be conceptualised as structured, system-level frameworks integrating digital technologies across the entire cancer care continuum from prevention and screening to diagnosis, treatment, survivorship, and population surveillance, as presented in a study by William et al. (17). Digital Oncology Frameworks can be categorised into several interrelated types as illustrated in Figure 1; where. (i) *Population-based cancer registry frameworks* collect, validate, and analyse incidence, mortality, and survival data at regional or national levels, supporting surveillance, epidemiological research, and policy planning (18); (ii) *Hospital-based oncology information management systems* manage oncology clinical workflows within oncology departments, including patient records, chemotherapy ordering, radiotherapy planning, imaging integration, and multidisciplinary coordination (19); (iii) *Tele-oncology and virtual tumour board platforms* leverage secure digital communication infrastructures to connect peripheral facilities with specialist centres, addressing workforce disparities and facilitating remote case review and collaborative treatment planning (20); (iv) Mobile health (mHealth) oncology tools support screening campaigns, patient navigation, adherence monitoring, symptom reporting, and appointment management (21); (v) *Cancer information hubs and data exchange platforms* are centralised repositories integrating registry data, clinical datasets, imaging archives, and research databases; and (vi) *Genomic and precision oncology platforms* integrate molecular diagnostics, genomic profiling, and bioinformatics pipelines into clinical decision-making (22). Although digital oncology initiatives are expanding across Africa, there is a limited systematic synthesis of their architectures, and maturity levels. Existing literature often focuses on individual technologies or country-specific implementations, without providing a continent-wide systems perspective. Consequently, policymakers, researchers, and implementers lack a consolidated understanding of current progress, structural gaps, and integration challenges. This scoping review hence presents a systematic review of digital oncology frameworks in Africa, categorises their architectural patterns, and maturity levels with the aim of identifying dominant system architectures, interoperability characteristics, implementation gaps, and digital maturity trends to inform the design, integration, scalability, and future development of equitable and interoperable digital oncology ecosystems across African health systems.

### 1.5 Digital maturity levels and architectural configurations of digital oncology frameworks

Assessing digital oncology frameworks requires not only categorising their typology but also characterising how they are structurally organised and how advanced their digital capabilities are. Two analytical dimensions are particularly relevant: architectural configuration (23) and digital maturity level (24). Architectural configuration refers to the structural design of a digital oncology framework; how its components are organised, how data flows between them, and how the system interfaces with external platforms. Five dominant architectural patterns are recognised in digital health systems literature (25). (i) *Centralised single-node architectures* that consolidate data capture, storage, and processing within a single institutional or national node, offering operational simplicity but limited scalability and interoperability; (ii) *Multi-site centralised architectures* extend a shared infrastructure across geographically distributed facilities, enabling consolidated reporting and economies of scale; (iii) *Hub-and-spoke architectures* connect a specialist centre with peripheral facilities through digital communication channels, supporting remote consultation, mentorship, and case review; (iv) *Federated network architectures* distribute data custody and analytical functions across multiple autonomous nodes operating under shared protocols and governance structures, balancing standardisation with data sovereignty; and (v) *Hybrid architectures* that combine elements of two or more of the above models to meet specific operational or regulatory requirements.

Digital maturity, on the other hand, refers to the level of sophistication, integration, and interoperability a framework has achieved (26). For this review, a three-level classification is applied. Level 1 denotes basic digitisation, characterised by paper-to-electronic data transition with minimal system integration or workflow automation. Level 2 denotes structured digital operation, characterised by centralised electronic data capture, defined clinical or surveillance workflows, and limited interoperability with external systems. Level 3 denotes integrated digital infrastructure, characterised by API-based or standards-compliant data exchange, cross-system interoperability, multi-site or nationally embedded operation, and structured output interfaces with regional or international reporting platforms. Artificial intelligence integration is assessed as a parallel dimension: Level 0 denotes no AI integration; Level 1 denotes rule-based automation or decision-support tools; and Level 2 and above denotes ML or advanced analytical AI components embedded within operational workflows. These two analytical dimensions, architectural configuration and digital maturity, provide the evaluative framework applied consistently across all six framework typologies in this review, enabling systematic cross-category comparison and the identification of structural patterns relevant to platform developers, health system planners, and policymakers.

## 2 Methodology

### 2.1 Study design

**Figure 2:**
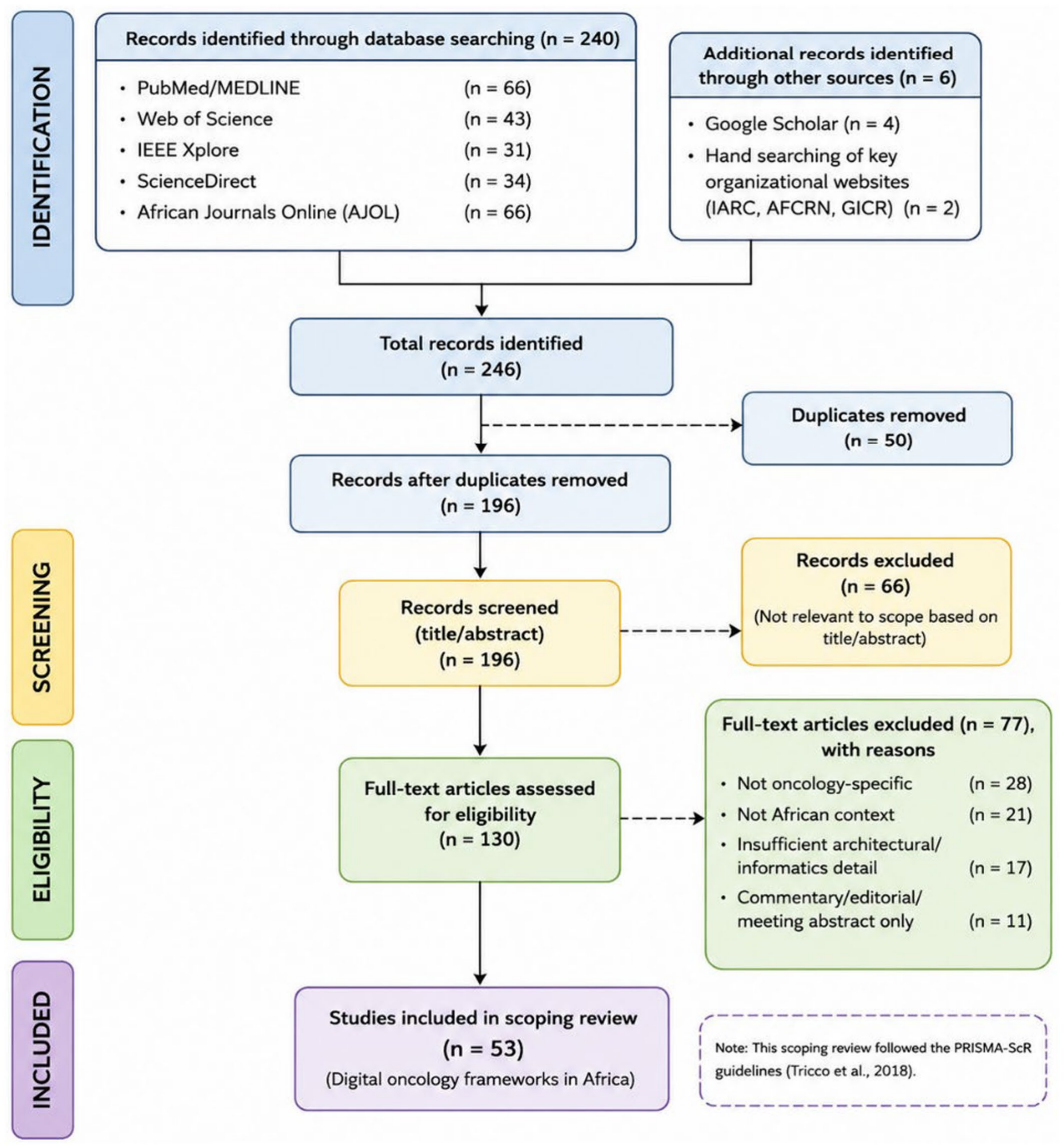
PRISMA-ScR flow diagram for research papers selection.

This study employed a scoping review methodology to systematically map and synthesise existing evidence on the architectural organisation of digital oncology frameworks in Africa. A scoping review was selected over a systematic review given the heterogeneity, emerging nature, and multidisciplinary scope of digital oncology spanning clinical information systems, telemedicine, mobile health, genomics, and public health surveillance where the primary objective is comprehensive architectural mapping rather than effect size estimation. The review followed the Arksey and O’Malley framework as refined by Joanna Briggs Institute (JBI) guidelines, and reporting was aligned with the PRISMA Extension for Scoping Reviews (PRISMA-ScR) to ensure transparency and reproducibility as shown in Figure 2. This review protocol was not prospectively registered because the study was conducted as an exploratory scoping review focused on architectural synthesis rather than intervention effectiveness.

### 2.2 Information sources and search strategy

A multi-database search was conducted across PubMed/MEDLINE, Web of Science, IEEE Xplore, ScienceDirect, and African Journals Online (AJOL). The search strategy combined terms across three conceptual domains: digital oncology interventions, architectural and informatics system characteristics, and the African geographic context. A representative search string was: (“digital oncology” OR “oncology information system” OR “cancer registry” OR “tele-oncology” OR “mHealth cancer” OR “precision oncology”) AND (“system architecture” OR “health information system” OR “interoperability” OR “platform design” OR “data exchange”) AND (“Africa” OR “sub-Saharan Africa” OR “low- and middle-income countries”). Searches were conducted without date restrictions and supplemented by citation tracking and manual searches of key organisational websites, including IARC, AFCRN, and GICR. The representative search strategy was adapted to the indexing structure and syntax requirements of each database. Full database-specific search strings, including Boolean operators, field tags, and search syntax, are provided in Supplementary File 1 to ensure reproducibility. The study selection process, including identification, screening, eligibility assessment, and final inclusion of studies, is presented in the PRISMA-ScR flow diagram (Figure 2).

### 2.3 Eligibility criteria and study selection

Studies were eligible if they described a digital oncology framework operating within Africa with sufficient detail to permit architectural characterisation. Eligible frameworks spanned population-based cancer registries, hospital-based oncology information systems, tele-oncology and virtual tumour board platforms, mobile health applications, cancer information hubs & data exchange platforms, and genomic or precision oncology frameworks. Studies were excluded if they reported frameworks unrelated to oncology, outside Africa, or lacking sufficient detail for analysis. Records were deduplicated and independently screened by two reviewers (WW, AW) at title/abstract and full-text stages, with discrepancies resolved by consensus (Table 1).

**Table 1:** Comprehensive summary of digital oncology frameworks included in the review.

Framework/system	Country/region	Category	Architecture	Digital maturity	AI integration	Primary function
1. Population-based cancer registries						
South Africa NCR	South Africa	PBCR	Centralised electronic (national DB)	Level 3	None	Surveillance, epidemiological research
Kampala Cancer Registry	Uganda	PBCR	Centralised (CanReg5)	Level 2	None	Surveillance, research
Nairobi Cancer Registry	Kenya	PBCR	Centralised (CanReg)	Level 2	None	Surveillance, research
Gharbiah Cancer Registry	Egypt	PBCR	Centralised electronic	Level 3	Limited	Surveillance, CI5 contribution
Casablanca Cancer Registry	Morocco	PBCR	Centralised electronic	Level 3	Limited	Surveillance, policy planning
Harare Cancer Registry	Zimbabwe	PBCR	Centralised (CanReg)	Level 2	None	Surveillance, HIV-assoc. malignancy research
Blantyre Cancer Registry	Malawi	PBCR	Centralised electronic	Level 2	Limited	Surveillance, research
Rwanda National Cancer Registry	Rwanda	PBCR	Centralised (national HIS)	Level 3	Limited	Surveillance, national implementation
Seychelles Cancer Registry	Seychelles	PBCR	Centralised national	Level 3	Limited	National surveillance
Ibadan Cancer Registry	Nigeria	PBCR	Centralised digital	Level 2	None	Surveillance, CI5 contribution
Kumasi Cancer Registry	Ghana	PBCR	Centralised electronic	Level 2	None	Surveillance, national burden estimation
Addis Ababa Cancer Registry	Ethiopia	PBCR	Centralised (CanReg)	Level 2	Limited	Surveillance, research
Bamako Cancer Registry	Mali	PBCR	Centralised electronic	Level 2	None	Surveillance, research
Antananarivo Cancer Registry	Madagascar	PBCR	Centralised electronic	Level 2	None	Surveillance, international reporting
2. Hospital-based oncology information systems						
Butaro Cancer Centre OIS (OpenMRS)	Rwanda	OIS	Centralised (facility-based)	Level 2	None	Diagnosis, treatment documentation, monitoring
NSIA-LUTH (Varian ARIA)	Nigeria	OIS	Departmental/vendor-integrated	Level 3	Limited	Radiotherapy record-and-verify
National Hospital Abuja (MOSAIQ)	Nigeria	OIS	Departmental/centralised	Level 3	Limited	Radiotherapy documentation, verification
Ocean Road Cancer Institute EMR	Tanzania	OIS	Facility-based centralised	Level 2	None	Diagnosis, treatment documentation
Uganda Cancer Institute (Varian ARIA)	Uganda	OIS	Centralised	Level 2	None	Diagnosis, treatment support
NCI Cairo HIS	Egypt	OIS	Integrated hospital HIS	Level 2	None	Oncology workflows, imaging archiving
Groote Schuur Hospital (ARIA)	South Africa	OIS	Departmental/vendor-integrated	Level 3	Limited	Radiotherapy documentation, device interop
Equra Healthcare (MOSAIQ cluster)	South Africa	OIS	Multi-site centralised	Level 3	Limited	Multi-site radiotherapy management
Kenyatta National Hospital (MOSAIQ)	Kenya	OIS	Departmental/centralised	Level 3	Limited	Radiotherapy documentation, workflow
3. Tele-oncology and virtual tumour board frameworks						
Africa Cancer Research & Control ECHO	Pan-African	Tele-oncology/VTB	Centralised coordination (Zoom)	Level 1	None (AI Level 0)	Tele-mentoring, case discussion, workforce dev.
E-learning cervical/breast cancer (French Africa)	Francophone Africa	Tele-oncology/VTB	Centralised teleconferencing	Level 1	None (AI Level 0)	Virtual tumour board, mentorship, capacity building
IGCS Global Curriculum ECHO VTB	Multi-country	Tele-oncology/VTB	Hub-and-spoke (ECHO model)	Level 1	None (AI Level 0)	Mentorship, clinical decision-making, gynecologic oncology
Zambia Gynecologic Oncology MTB Platform	Zambia	Tele-oncology/VTB	Centralised digital dashboard	Level 2	None (AI Level 0)	MDT case discussion, referral tracking, patient navigation
Butaro Telepathology Triage System	Rwanda	Tele-oncology/VTB	Hub-and-spoke (remote pathology)	Level 2	None (AI Level 0)	Remote pathology, diagnostic triage
Rwanda National MDT Tumour Boards	Rwanda	Tele-oncology/VTB	Centralised institutional	Level 2	None (AI Level 0)	Multidisciplinary case review, treatment planning
Virtual Ultrasound Training (Weimer 2024)	Sub-Saharan Africa	Tele-oncology/VTB	Teleconferencing	Level 1	None (AI Level 0)	Diagnostic capacity building, image review
4. Mobile health (mHealth) oncology frameworks						
SMS Cervical Cancer Screening – Ghana	Ghana	mHealth	Centralised SMS delivery	Level 1	None (AI Level 0)	Screening uptake, awareness (cervical cancer)
SMS HPV Follow-up Reminders – Tanzania	Tanzania	mHealth	Centralised SMS scheduling	Level 1	None (AI Level 0)	Follow-up attendance after HPV + screening
Educative/Reminder SMS – Tanzania	Tanzania	mHealth	Centralised one-way SMS	Level 1	None (AI Level 0)	Behavioural prompting, cervical cancer screening
CCPPZ Mobile Messaging – Zambia	Zambia	mHealth	Centralised bulk SMS	Level 1–2	None (AI Level 0)	Demand generation, screening uptake
WHO mCervicalCancer Initiative	Multi-country	mHealth	Standardised SMS/app architecture	Level 1–2	None (AI Level 0)	Prevention education, screening, follow-up adherence
Smartphone Cervicography (EVA) – Zambia	Zambia/SSA	mHealth	Clinic-centred, optional telehealth	Level 1–2	None (AI Level 0)	Mobile imaging, cervical screening, triage
SMS/Call HPV Vaccination Reminders – Uganda	Uganda	mHealth	Centralised outbound messaging	Level 2	None (AI Level 0)	HPV vaccination adherence, primary prevention
mPCL Palliative Care Platform	Tanzania	mHealth	Centralised cloud/web	Level 2	None	Palliative care, symptom monitoring, follow-up
PCAU mHealth Surveillance – Uganda	Uganda	mHealth	Centralised data collection/analytics	Level 2–3	None (AI Level 0)	Palliative care reporting, national surveillance
Mobile Psychoeducation – Nigeria	Nigeria	mHealth	Mobile delivery (app)	Level 1–2	None (AI Level 0)	Psychoeducation, breast cancer chemotherapy support
5. Cancer information hubs and data exchange frameworks						
Global Cancer Observatory (GCO/GLOBOCAN)	Global (IARC)	Data Hub/Exchange	Centralised analytics platform	Level 3	Limited	Surveillance, policy planning, research
Cancer Incidence in Five Continents (CI5/CI5plus)	Global (IARC)	Data Hub/Exchange	Centralised editorial/quality system	Level 3	None	Epidemiology benchmarking, incidence reporting
African Cancer Registry Network (AFCRN)	Pan-African	Data Hub/Exchange	Coordination/networking hub	Level 2	None	Registry strengthening, surveillance capacity
GICR Sub-Saharan Africa Hub	Pan-African	Data Hub/Exchange	Hub-based coordination	Level 2	None	Registry development, training, standardisation
CanReg5 Platform	Pan-African	Data Hub/Exchange	Open-source national registry tool	Level 2	None	Data capture, validation, management, analysis
DHIS2–IARC Cancer Registry Toolkit	Multi-country	Data Hub/Exchange	National HIS-integrated	Level 3	None	Interoperable registry data, national HIS integration
DHIS2 Tracker Cervical Cancer (SUCCESS)	Burkina Faso, Côte d’Ivoire	Data Hub/Exchange	Centralised national tracker	Level 3	None	Screening-diagnosis linkage, follow-up
ICCP Portal	Global	Data Hub/Exchange	Centralised web repository	Level 2	None	Cancer control policy knowledge hub
6. Genomic and precision oncology frameworks						
H3Africa Initiative	Pan-African	Genomic/Precision	Networked (distributed sites)	Level 3	Limited	Biorepositories, genomic pipelines, cancer genomics
H3ABioNet	Pan-African	Genomic/Precision	Federated multi-node	Level 3	Limited	Bioinformatics, genomic data analysis capacity
AFBRECANE Study	Nigeria	Genomic/Precision	Multi-site centralised	Level 3	Limited	Female breast cancer genomics/GWAS, epidemiology
MADCaP Consortium	Multi-country (4)	Genomic/Precision	Multi-country standardised	Level 3	Limited	Prostate cancer genetic epidemiology, biospecimen
African Pharmacogenomics Network (APN)	Pan-African	Genomic/Precision	Network-based	Level 3	Limited	Pharmacogenomics, precision drug use in oncology

PBCR, Population-Based Cancer Registry; OIS, Oncology Information System; VTB, Virtual Tumour Board; mHealth, Mobile Health; DML, Digital Maturity Level; AI, Artificial Intelligence; SSA, Sub-Saharan Africa; HIS, Health Information System; IARC, International Agency for Research on Cancer.

### 2.4 Data extraction and synthesis

A standardised, *a priori* extraction template was used to capture framework characteristics, including country of implementation, framework category, cancer care continuum coverage, architectural topology, interoperability characteristics, digital maturity level, and AI integration status.

Digital maturity was operationalised using predefined structural criteria adapted for digital oncology systems in low- and middle-income settings: Level 1 represented basic standalone digitisation with minimal interoperability; Level 2 represented centralised electronic systems with structured workflows but limited cross-system integration; and Level 3 represented interoperable, multi-site or nationally embedded architectures with API- or standards-based data exchange. Standalone SMS reminder systems were typically classified as Level 1–2, whereas registry systems integrated into national health information infrastructures were classified as Level 3.

AI integration was similarly categorised as: Level 0 (no AI functionality or conventional digital workflows only), Level 1 (rule-based automation without machine learning), and Level 2 or higher (machine learning, predictive analytics, computer vision, natural language processing, or adaptive analytical models). Conventional dashboards, teleconferencing systems, and SMS platforms without adaptive learning capability were classified as AI Level 0.

Architectural characterisation was the primary analytical focus and included dominant topology (centralised, multi-site centralised, federated, hub-and-spoke, or hybrid), data flow directionality, interoperability interface type, and integration with national health information systems. Formal inter-rater reliability statistics were not calculated because classification involved qualitative architectural interpretation rather than quantitative measurement.

## 3 Results

### 3.1 Population-based cancer registry frameworks in Africa

Population-based cancer registry frameworks are foundational to cancer control in Africa, providing systematic collection, analysis, and reporting of cancer incidence, mortality, and survival data at regional and national levels. These systems support epidemiological surveillance, inform policy and planning, and enable monitoring of cancer trends over time. In resource-constrained settings, they are critical for generating reliable data to guide prevention, screening, and treatment strategies, while also facilitating research and international comparisons. *The South Africa National Cancer Registry (NCR)* (27), administered by the National Department of Health, is one of the most comprehensive population-based cancer registry frameworks on the continent. The registry supports surveillance and epidemiological research and relies on centralised electronic pathology reporting systems integrated into a national database. Although operationally mature, interoperability with hospital electronic health records and AI integration remains limited (28). Schonfeld et al. used the registry to document hematologic malignancies in South Africa, 2000–2006 (29). *The Kampala Cancer Registry* operates as a regional registry in Uganda (30). The registry utilises the IARC-supported CanReg5 platform for electronic data capture and focuses on surveillance and research across multiple cancer types. Its centralised architecture positions it at Digital Maturity Level 2; however, with no AI integration (31). This has been used by Bukirwa et al. to document the trends in the incidence of cancer in the population of Kyadondo County, Uganda, for a period of 25 years (1991–2015). The Nairobi Cancer Registry, coordinated through academic institutions and the Kenya Medical Research Institute, functions as an urban population-based registry in East Africa (32). It employs centralised digital data entry using CanReg and supports surveillance and research functions, but does not directly integrate with national EHR infrastructures. AI integration is absent, and interoperability standards are not formally embedded; maturity is classified at Level 2. *The Gharbiah Cancer Registry is* one of the most internationally cited African cancer registries (33). Functioning as a regional registry with structured electronic data systems, it has contributed extensively to global cancer epidemiology through cancer incidence in five continents (CI5) publications (34). Its centralised architecture positions it at Digital Maturity Level 3, but with limited AI integration. *The Casablanca Cancer Registry in Morocco* is a regional cancer registry in North Africa and is integrated into the national cancer control strategy (35). The registry supports surveillance and policy planning and has contributed to understanding cancer epidemiology in urban populations. The registry is classified at Digital Maturity Level 3 but with limited AI integration and formal interoperability standards (35). *The Harare Cancer Registry in Southern Africa* operates as a regional cancer registry and has generated important epidemiological data on HIV-associated malignancies (36). It utilises centralised electronic data systems, primarily through CanReg, and digital maturity is Level 2. *The Blantyre Cancer Registry* in Southern Africa utilises electronic data entry systems and hospital-based reporting mechanisms to capture incidence data for surveillance and research purposes (37). The registry operates at Digital Maturity Level 2 with limited AI capabilities. *The Rwanda National Cancer Registry* operates within the country’s centralised health information management system and reflects one of the more digitally aligned registry models in East Africa (38). Although AI integration is limited, the hybrid centralised architecture and national implementation scale position it at Digital Maturity Level 3. *The Seychelles Cancer Registry* is fully national, and it demonstrates relatively high data completeness and quality (39). Its scale allows efficient surveillance coverage, positioning it at Digital Maturity Level 3. *The Ibadan Cancer Registry* in West Africa is one of the longest-standing cancer registries in the region (40). It employs centralised digital data capture platforms and contributes to CI5 publications. Operating at a regional scale, it supports surveillance and research and is classified at Digital Maturity Level 2. *The Kumasi Cancer Registry* functions as a regional PBCR in West Africa and has contributed to national cancer burden estimation (41). Utilising centralised electronic data entry systems, it supports surveillance and research, and digital maturity is Level 2. *The Addis Ababa Cancer Registry* utilises CanReg-based centralised systems to capture cancer incidence data. Digital maturity is Level 2 with limited AI integration (42). The Bamako Cancer Registry serves as a regional cancer registry in West Africa (43). Using centralised electronic data capture, it operates at Digital Maturity Level 2. *The Antananarivo Cancer Registry* in Madagascar operates as a regional cancer registry with centralised electronic data systems (44). It supports surveillance, research functions and contributes to international cancer incidence datasets, and digital maturity is Level 2.

### 3.2 Hospital-based oncology information systems in Africa

Hospital-based oncology information systems (OIS) are central to the digital management of cancer care within clinical settings, supporting the coordination of diagnosis, treatment, and follow-up services. These systems integrate patient records, chemotherapy and radiotherapy workflows, imaging, laboratory data, and multidisciplinary documentation to enable efficient and standardised care delivery. In the African context, OIS are increasingly being implemented in tertiary and referral centres to improve clinical decision-making, reduce fragmentation of care, and enhance data availability for monitoring and quality improvement. Their importance lies in strengthening facility-level cancer management while providing a foundation for broader digital oncology integration. The *Butaro Cancer Centre of Excellence in Rwanda* operates a hospital-based oncology information system built on *OpenMRS* (45) to support diagnosis, treatment documentation, and service monitoring. The system integrates structured oncology modules within a centralised architecture, and digital maturity aligns with Level 2 (workflow integration). Validation studies have shown improved cancer care (46). At NSIA-LUTH (West Africa), the Varian ARIA oncology information system supports radiotherapy record-and-verify workflows within a tertiary cancer centre (47). The architecture is departmentally centralised and vendor-integrated. Digital maturity is Level 3 within radiotherapy operations. Outcome studies have shown enhanced treatment safety through record and verify integration (48). The National Hospital Abuja utilises the MOSAIQ oncology information system to manage radiotherapy documentation and verification processes (49). This facility-level framework supports treatment-phase coordination within centralised departmental architecture, and digital maturity is Level 3 in radiotherapy services. *Ocean Road Cancer Institute in Tanzania* operates an institutional EMR to support diagnosis and treatment documentation within its national cancer centre. The architecture is facility-based and centralised with digital maturity at Level 2. Outcome studies have shown improved record availability and workflow documentation (50). The Uganda Cancer Institute utilises Varian ARIA based platform to support diagnosis and treatment (51). Architecture is centralised with limited interoperability and digital maturity is Level 2. The National Cancer Institute Cairo implemented an integrated hospital information system with digitised medical records and imaging archiving to support oncology workflows (52). The framework supports diagnosis and treatment documentation, and digital maturity is Level 2. Groote Schuur Hospital integrates Varian ARIA within radiotherapy workflows to support treatment documentation and device interoperability. The architecture is departmental and vendor-integrated and at digital maturity Level 3 within radiotherapy services. Outcomes assessments have shown improved workflow integration and treatment verification (53). Equra Healthcare utilises a multi-site MOSAIQ cluster to manage radiotherapy services across facilities, demonstrating centralised multi-hospital oncology informatics architecture. Digital maturity is Level 3 and outcomes include multi-site data consolidation and operational integration. Similarly, Kenyatta National Hospital employs MOSAIQ to support radiotherapy documentation and workflow coordination. The framework is departmental and centralised and digital maturity is Level 3 within radiotherapy services.

### 3.3 Tele-oncology and virtual tumour board frameworks

Tele-oncology and virtual tumour board frameworks enable remote delivery of oncology services and multidisciplinary collaboration through digital communication technologies. These frameworks connect peripheral or resource-limited facilities with specialised cancer centres, allowing clinicians to access expert consultations, review diagnostic data, and jointly develop treatment plans in real time. Virtual tumour boards, in particular, facilitate coordinated decision-making among oncologists, radiologists, pathologists, and surgeons across geographic boundaries. In the African context, where specialist shortages and uneven distribution of oncology services are common, these frameworks are essential for improving access to expertise, enhancing quality of care, and supporting capacity building. Their importance lies in reducing disparities in cancer management while strengthening integrated and collaborative care delivery systems. Kabukye et al. describe the *Africa Cancer Research and Control ECHO* as a continental tele-mentoring and case-discussion platform hosted via Zoom and coordinated through a multi-country steering structure, with participation reported across multiple African countries. The framework functions primarily as a virtual tumour board/telementoring model supporting workforce development and evidence-based decision-making, using video-conferencing and structured case-based learning within a centralised coordination model; AI integration is not reported (AI Level 0). Outcomes include ongoing participation, collaboration outputs, and capacity-building products (54). Domgue et al. document the *Enhancing cervical and breast cancer training in Africa with e-learning*, implemented across French-speaking African countries as a virtual mentorship and case-discussion platform using video conferences “in the form of virtual tumour boards.” The framework targets women’s cancers, uses routine teleconferencing and structured curricula. Reported outcomes include participation and satisfaction metrics as proxies for workforce capacity-building impact (55). The International Gynecologic Cancer Society (IGCS) describes a structured *Project ECHO virtual tumour board* component embedded within its Global Curriculum and Mentorship Programme, enabling recurring multidisciplinary case review and didactics between local trainees and international faculty (56). The programme uses the hub-and-spoke Project ECHO model to support mentorship, clinical decision-making, and capacity building in gynecologic oncology across multiple training sites. Pattee et al. describe the implementation of a virtual interactive system supporting gynecologic oncology multidisciplinary tumour boards in Zambia, integrating digital dashboards and referral tracking tools to enhance patient navigation and care coordination. The platform enabled structured multidisciplinary case discussions and centralised tracking of clinical decisions, demonstrating the feasibility of digital tumour board systems in low-resource oncology settings (57). Mpunga et al. describe the implementation of a telepathology triage system at Butaro District Hospital in Rwanda, designed to accelerate accurate cancer diagnosis. The platform enabled digital transmission of pathology slide images to remote specialist pathologists for interpretation, supporting diagnostic decision-making within a structured triage workflow. Using a hub-and-spoke model of remote consultation, the intervention demonstrated the feasibility of telepathology services in low-resource settings and improved diagnostic turnaround time for cancer patients (58). Rwanda has implemented multidisciplinary tumour boards as a routine component of cancer service delivery, bringing together oncologists, surgeons, radiologists, pathologists, and other specialists to review complex cases and develop consensus treatment plans. These tumour board meetings facilitate collaborative decision-making and improve coordination of oncology care across institutions (59). Weimer et al. describe a proof-of-concept ultrasound training programme that combined onsite instruction with recurring virtual case discussions structured similarly to tumour board meetings. The programme used teleconferencing platforms to review ultrasound images and clinical cases, enabling remote expert feedback on diagnostic image acquisition and interpretation. Over 24 virtual sessions, measurable improvements in ultrasound image quality were observed, demonstrating the potential of structured virtual review mechanisms to strengthen diagnostic capacity in low-resource settings (60).

### 3.4 Mobile health (mHealth) oncology frameworks

Mobile health (mHealth) oncology frameworks leverage widespread mobile phone penetration to support cancer prevention, screening, diagnosis, treatment adherence, and follow-up care across diverse settings. These frameworks utilise technologies such as SMS, mobile applications, and voice-based systems to deliver health education, patient reminders, symptom monitoring, and care navigation, particularly in resource-constrained and geographically dispersed populations. In Africa, mHealth plays a critical role in bridging access gaps, enhancing patient engagement, and strengthening continuity of care by connecting individuals to health services in real time. Its importance lies in its scalability, cost-effectiveness, and ability to integrate with existing health systems, making it a key enabler of equitable and decentralised cancer care delivery. In urban Ghana, a culturally tailored one-way SMS mHealth intervention was developed to improve cervical cancer screening uptake. The intervention emphasised stakeholder-informed message co-design, cultural and linguistic tailoring, and feasibility of delivery through routine telecom networks (61). In Tanzania, an mHealth follow-up framework used one-way SMS reminders to improve attendance at scheduled follow-up screening among women who tested HPV positive. The platform is SMS-based with centralised scheduling and minimal interoperability needs and outcomes assessed appointment attendance at extended follow-up (62). An implementation study in Tanzania examined educative and reminder SMS for cervical cancer screening, finding high acceptability even where knowledge gains were modest or not the primary target of measurement. The technology is one-way SMS with centralised delivery and the framework’s value is primarily behavioural prompting and engagement (63). Zambia’s Cervical Cancer Prevention Programme (CCPPZ) integrates mobile messaging as a demand-generation strategy to support cervical cancer screening uptake. Within this framework, one-way bulk SMS is used to enhance awareness and prompt both initial and repeat screening, operating along the awareness-to-screening continuum. Reported outcomes include increased screening attendance, although challenges persist in terms of uneven message reach, limited interactivity, and health system capacity to absorb demand (64). The World Health Organisation’s mCervicalCancer initiative provides a structured mobile health (mHealth) framework for cervical cancer control, leveraging mobile channels such as SMS and mobile applications to support prevention education, screening uptake, and follow-up adherence. Rather than a single-country implementation, the initiative functions as a standardised implementation architecture, offering guidance on message content, delivery workflows, and monitoring systems to support national programmes. Operating at AI Level 0, the framework emphasises scalability, standardisation, and integration into existing health systems (65). In Zambia and other sub-Saharan African settings, cervical cancer screening programmes have increasingly incorporated smartphone-based digital cervicography systems, including enhanced visual assessment (EVA), as mobile imaging and telehealth-enabled approaches for screening and triage. These systems integrate smartphone optics, digital image capture, and enhanced visualisation to support clinical decision-making, with optional remote expert review in telemedicine workflows. Typically deployed within clinic-centred architectures, they operate at AI Level 0 with digital maturity ranging from Level 1 to 2 (66). In Uganda, a randomised evaluation assessed the effectiveness of SMS and automated phone call reminders in improving the timeliness of human papillomavirus (HPV) vaccination among adolescent girls. The intervention employed a centralised scheduling system with outbound messaging and voice call reminders to support adherence to vaccination schedules, operating at AI Level 0 with a digital maturity of Level 2. The study demonstrated that mobile-based reminders improved timely completion of HPV vaccination compared to standard practice, highlighting the effectiveness of low-cost digital communication tools in strengthening primary prevention pathways (67). In Tanzania, *mPCL* is a smartphone/web-based mHealth framework designed to extend specialist palliative care through shared data between specialists and local health workers, supporting symptom assessment (notably pain), communication, and follow-up. Architecture is a patient/local provider app and specialist-facing interface, typically centralised cloud/web backend and at maturity Level 2 (tested/operationalised in study settings) (68). The Palliative Care Association of Uganda (PCAU) operationalised an mHealth surveillance framework where providers submit routine palliative care data via mobile app-based reporting, enabling national aggregation and feedback to stakeholders. Architecture is centralised data collection/analytics and maturity Level 2–3 (multi-site routine reporting) (69). In Nigeria, a feasibility study assessed perceived feasibility and usefulness of a mobile psychoeducation intervention to support women with breast cancer on chemotherapy. The framework uses mobile delivery of structured education and supportive content and the maturity Level is 1–2. All participants indicated that a mobile psychoeducation intervention was acceptable, stating it could provide psychological and informational support (70).

### 3.5 Cancer information hubs and data exchange frameworks

Cancer information hubs and data exchange frameworks constitute a critical layer of digital oncology infrastructure, enabling the aggregation, integration, and dissemination of cancer-related data across clinical, public health, research, and community domains. These frameworks consolidate data from cancer registries, hospital information systems, imaging platforms, laboratories, and research databases into centralised or federated architectures that support interoperability and coordinated data use. In parallel, cancer information hubs designed for the public, such as patient portals, awareness platforms, and digital information services, play an equally important role by providing accessible, reliable, and culturally appropriate cancer information. These platforms enhance health literacy, support patient navigation, and build trust in health systems by reducing misinformation and improving transparency. Some of the available cancer information hubs and data exchange platforms include. The *Global Cancer Observatory (GCO)* (71) is an IARC-managed, web-based cancer information hub that provides standardised estimates of cancer incidence, mortality, and projections for Africa and all regions It supports surveillance, policy planning, research and operates as a centralised analytics platform with downloadable country profiles and interactive visualisation tools; interoperability is “export-oriented” (data access rather than bidirectional exchange). *The Cancer Incidence in Five Continents (CI5) and CI5plus* function as global data exchange and benchmarking platforms, publishing comparable incidence data and time trends submitted by population-based cancer registries, including African registries that meet quality criteria. The platform supports surveillance, epidemiology, benchmarking, and the architecture is centralised with rigorous editorial/quality review, enabling cross-country comparability; interoperability is defined by standardised coding/metadata rather than HL7/FHIR-type interfaces (34). *The African Cancer Registry Network (AFCRN)* (72) operates as a continental coordination and technical-support hub for strengthening population-based cancer surveillance across sub-Saharan Africa for surveillance capacity-building, research enabling. The platform aggregates registry resources, supports standardisation, and facilitates technical assistance and networking, rather than serving as a transactional health information exchange. *The Global Initiative for Cancer Registry Development (GICR) hubs*, including the Sub-Saharan Africa hub, function as a knowledge and capacity hub supporting registry development through training, technical assistance, and standard operating approaches. The architecture is hub-based coordination with learning and support infrastructure; interoperability is indirectly supported through standardisation and tools rather than data exchange pipelines. *CanReg5* (73) is the dominant open-source platform used by many African PBCRs to input, validate, manage, and analyse registry data, enabling standardisation and comparability across registries. The framework is mainly used for surveillance and research. While not a “hub” itself, it is a foundational data infrastructure that supports harmonised datasets for national aggregation and international reporting. *DHIS2–IARC cancer registry toolkit initiative*, a collaboration between IARC and the DHIS2 ecosystem, aims to scale interoperable cancer registry capabilities by enabling countries to collect, manage, and analyse cancer data within national health information systems, improving compatibility with international reporting standards. This is explicitly a data exchange/integration framework (national HIS + cancer registry functions), with expected interoperability uplift and national embedding. *DHIS2 Tracker cervical cancer programme*; a cervical cancer configuration implemented through the SUCCESS project in *Burkina Faso and Côte d’Ivoire* functions as a programmatic data exchange framework linking facilities and laboratories, with notifications that improve return of results and follow-up adherence, and it supports screening, diagnosis linkage and follow-up. Architecture is centralised national/programme tracker with facility-lab reporting workflows; interoperability is internal to DHIS2 with exportable reporting. The *International Cancer Control Partnership (ICCP) portal* serves as a global–regional policy knowledge hub, hosting national cancer control plans and providing an interactive map that assesses comprehensiveness across cancer control domains. Architecture is centralised web repository and analytics; interoperability is document/search-based.

### 3.6 Genomic and precision oncology frameworks in Africa

Genomic and precision oncology frameworks represent an emerging frontier in digital cancer care in Africa, focusing on the integration of molecular diagnostics, genomic sequencing, and bioinformatics into clinical decision-making. These frameworks enable the characterisation of tumour biology at the genetic and molecular levels, allowing for more accurate diagnosis, risk stratification, and personalised treatment selection. In the African context, where genetic diversity is high and underrepresented in global datasets, such frameworks are particularly important for generating locally relevant evidence and improving the effectiveness of targeted therapies. The *Human Heredity and Health in Africa (H3Africa)* (74) initiative provides a continent-scale precision oncology enabling framework through coordinated biorepositories, harmonised consent/ELSI practices, and standardised genomic pipelines that support cancer and non-cancer genomics across multiple African countries. The architecture is networked (distributed sites with shared standards) and interoperability is primarily standards/protocol-based rather than clinical HL7/FHIR exchange. *H3ABioNet (pan-African bioinformatics network enabling precision oncology analytics)* (75) is a pan-African bioinformatics network established to enable genomic data analysis capacity (including cancer genomics) through distributed nodes, shared pipelines, training, and access to computational infrastructure. The framework is federated by design (multi-node), with workflow interoperability achieved via shared tools and QC standards. The *African Female Breast Cancer Epidemiology (AFBRECANE)* (76) study is a structured genomic and precision-oncology research framework in Nigeria that integrates multi-site clinical recruitment with epidemiologic characterisation and genomic analyses (e.g., GWAS), explicitly linked to population-based cancer registry ascertainment. The architecture is multi-site with centralised data harmonisation; interoperability is achieved through protocolized data models across sites. The *Men of African Descent and Carcinoma of the Prostate (MADCaP)* consortium operationalizes a multicenter genetic epidemiology framework for prostate cancer across four African countries, standardising recruitment, biospecimen collection, and genomic analysis to address aetiology and outcomes in men of African ancestry. The architecture is multi-country with standardised protocols; interoperability is consortium-standard driven (77). The *African Pharmacogenomics Consortium/Network (APN)* (78) provides a continent-wide precision medicine framework that is highly relevant to oncology through pharmacogenomic evidence generation for safer and more effective drug use in African populations. The architecture is network-based, interoperability is protocol and training-driven.

### 3.7 Quantitative synthesis of included frameworks

A total of 53 digital oncology frameworks were identified across six functional categories and more than 30 African countries or sub-regional settings. Population-based cancer registries constituted the largest category (*n* = 14; 26.4%), followed by mHealth frameworks (*n* = 10; 18.9%), hospital-based oncology information systems (*n* = 9; 17.0%), cancer information hubs and data exchange platforms (*n* = 8; 15.1%), tele-oncology and virtual tumour board frameworks (*n* = 7; 13.2%), and genomic and precision oncology frameworks (*n* = 5; 9.4%). Digital maturity assessments revealed that 36 frameworks (67.9%) were classified at Level 2 or below, indicating limited advanced interoperability across the landscape. Genomic frameworks demonstrated the highest aggregate maturity, with all five (100%) at Level 3, followed by OIS (6/9; 66.7%). Conversely, all tele-oncology and mHealth frameworks remained at Level 1 or 2, reflecting the low-complexity communication architectures such as one-way SMS and video-conferencing on which these interventions predominantly rely. No framework demonstrated full or substantial AI integration. Forty frameworks (75.5%) reported no AI integration (AI Level 0), while 13 (24.5%) demonstrated only limited features such as structured analytics or basic decision-support tools. AI Level 0 was universal among tele-oncology and mHealth frameworks, and predominant among population-based cancer registries (11/14; 78.6%). These findings indicate that AI integration across digital oncology infrastructure in Africa remains low, with no framework currently operating at a level consistent with validated, clinically embedded artificial intelligence.

## 4 Discussion

This scoping review identified six categories of digital oncology framework architectures operating across Africa: population-based cancer registry frameworks, hospital-based oncology information systems, tele-oncology and virtual tumour board frameworks, mobile health oncology frameworks, cancer information hubs and data exchange frameworks, and genomic and precision oncology frameworks. While prior literature has documented the existence and outputs of many of these frameworks individually, the architectural perspective on how each framework is structured, how its components are organised and interconnected, and what that structure implies for scalability, interoperability, and replicability has received comparatively little systematic attention. This discussion addresses that gap by examining the dominant architectural patterns within each framework category, interpreting their implications for platform developers, and identifying cross-cutting architectural lessons and deficits relevant to the design of future digital oncology systems in Africa.

### 4.1 Population-based cancer registries: centralised surveillance architectures

The architectural pattern most consistently observed across African population-based cancer registries (PBCRs) is centralised, single-node data aggregation, predominantly implemented through the CanReg5 platform (79). In this architecture, cancer incidence data from hospitals, pathology laboratories, and clinical services are submitted to a single registry centre for entry, validation, deduplication, and storage. This pattern is evident across nine registries, including the Kampala, Nairobi, Harare, Blantyre, Addis Ababa, Bamako, Antananarivo, Ibanda, and Kumasi Cancer Registries all operating at Digital Maturity Level 2 (80). Its dominance reflects CanReg’s status as the IARC-endorsed standard: open-source, low-infrastructure, and compatible with international reporting frameworks such as CI5. However, centralised single-node models are inherently dependent on manual data submission workflows, creating bottlenecks in completeness and timeliness, and lack native interoperability interfaces with hospital EHR systems or national health platforms, requiring non-standardised file transfers rather than structured API-based exchange. A more advanced sub-group, the Rwanda, Gharbiah, Casablanca, and Seychelles registries operate at Digital Maturity Level 3 within hybrid or nationally embedded architectures. Rwanda is particularly instructive: its integration within the national health information management system embeds cancer surveillance within broader digital health infrastructure rather than maintaining a standalone vertical system, providing a structural pathway towards interoperability, automated data feeds, and national analytics. For developers, three architectural insights follow: CanReg5-based architectures remain appropriate as a foundational layer but must be complemented by interoperability modules compatible with DHIS2, OpenMRS, or HL7/FHIR standards; the Rwanda model demonstrates that embedding registry functions within national HIS produces greater maturity and sustainability than parallel vertical systems (38); and future platforms should adopt a modular, API-first architecture that separates data capture, validation, storage, and analytics layers to enable incremental upgrades, including eventual AI integration, without wholesale system replacement.

### 4.2 Hospital-based oncology information systems (OIS): modular but siloed clinical architectures

Hospital-based OIS in Africa exhibit a clear architectural split between vendor-integrated proprietary platforms and open-source or institutional EMR solutions. Vendor-integrated platforms, principally Varian ARIA and Elekta MOSAIQ, are deployed across NSIA-LUTH, National Hospital Abuja, Groote Schuur Hospital, Equra Healthcare, and Kenyatta National Hospital, achieving Digital Maturity Level 3 within radiotherapy record-and-verify workflows through closed-loop departmental architectures that tightly integrate treatment planning systems, linear accelerator hardware, and clinical documentation (81). However, these platforms operate within proprietary data models that resist external interoperability, requiring costly middleware or extract-transform-load pipelines for exchange with registries or national health platforms, and creating structural vulnerability through vendor dependency, a significant risk in constrained African procurement environments. The contrasting open-source model, adopted by Butaro Cancer Centre’s OpenMRS-based OIS, offers open data models, active API development, and FHIR compatibility, achieving Digital Maturity Level 2 while providing a more interoperable and adaptable foundation for integration with registries, laboratory systems, and national health platforms. A third model Equra Healthcare’s multi-site MOSAIQ cluster is particularly instructive: its centralised server infrastructure supporting OIS functions across multiple facilities reduces per-facility costs, enables consolidated reporting, and creates economies of scale, offering a replicable blueprint for national or regional oncology information systems serving geographically distributed facilities. Three architectural insights follow for OIS developers. First, the choice between vendor-integrated and open-source architectures is a fundamental trade-off between departmental performance depth and system-level interoperability breadth. Second, multi-site centralised architectures should be prioritised in national infrastructure planning. Third, regardless of platform choice, OIS architectures must include formally specified interoperability interfaces with cancer registry systems, laboratory platforms, and national EHR infrastructure to support longitudinal patient data and population-level surveillance.

### 4.3 Tele-oncology: distributed, hub-and-spoke architectures

Tele-oncology and virtual tumour board frameworks in Africa converge on a single dominant architectural model: the hub-and-spoke configuration (82), in which specialist centres provide expertise, case review, and mentorship to peripheral facilities through digital communication channels. This architecture is operationalised across multiple scales, from continental platforms such as Africa Cancer Research and Control ECHO and the IGCS Global Curriculum and Mentorship Programme, to national models such as Rwanda’s multidisciplinary tumour board, to facility-level implementations such as Zambia’s digital tumour board and the Butaro telepathology platform. Despite variation in scope, all these platforms share a common structural logic: centralised hub coordination combined with distributed spoke participation, delivered through a deliberately lightweight technological layer; videoconferencing, structured case templates, and referral dashboards that prioritise accessibility over sophistication and enables participation without specialised hardware or high-bandwidth connectivity. Two architectural variants merit particular attention. Rwanda’s national tumour board has evolved from a project-based platform to a routinely institutionalised care coordination architecture embedded within standard clinical pathways, representing the transition from programme-level to system-level integration. Butaro’s telepathology platform adds an asynchronous layer, decoupling pathology review from synchronous meeting schedules through digital image transmission, a variant particularly relevant where real-time connectivity or specialist availability is constrained. For developers, four architectural recommendations follow: the hub-and-spoke model should be the default framework given its demonstrated feasibility and alignment with Africa’s specialist distribution; platforms must accommodate both synchronous and asynchronous workflows to ensure continuous operation regardless of connectivity; the Rwanda model sets the long-term architectural goal of system-level embedding within national clinical pathways rather than standalone operation; and referral tracking, outcome documentation, and longitudinal follow-up modules should be treated as structural requirements, not optional enhancements (83).

### 4.4 mHealth oncology frameworks: highly scalable but shallow architectures

mHealth oncology frameworks in Africa are architecturally dominated by centralised, unidirectional outbound SMS systems, in which pre-scheduled health education, appointment reminders, or behavioural prompts are pushed to patient phone numbers without structured mechanisms for response, bidirectional exchange, or clinical feedback (84). This architecture, operationalised through bulk SMS aggregator platforms requiring minimal infrastructure and no smartphone dependency, is deployed across cervical cancer screening programmes in Ghana, Tanzania, and Zambia; HPV vaccination adherence interventions in Uganda; and palliative care follow-up in Tanzania and Uganda. Its scalability, feature-phone compatibility, and demonstrated effectiveness in improving screening attendance, vaccination completion, and appointment adherence validate the proposition that population-level behaviour change can be meaningfully supported through lightweight, ubiquitous communication channels. However, one-way architectures impose a structural ceiling: the inability to capture patient-generated data symptom reports, adherence signals, and side effect notifications fundamentally constrains capacity for clinical monitoring, personalised care, or adaptive intervention. More advanced implementations illustrate the architectural evolution pathway beyond this ceiling. Tanzania’s mPCL platform enables bidirectional symptom data exchange between patients, community health workers, and remote specialists, while the Palliative Care Association of Uganda’s mobile reporting system supports structured multi-site data submission to a centralised analytics platform, both operating at Digital Maturity Level 2–3. The Zambia smartphone-based digital cervicography system extends the architectural scope further, integrating mobile imaging and clinical decision support within a clinic-centred workflow, providing a structural foundation for future AI-enabled image analysis. For developers, the architectural implication is a clear functional stratification: one-way SMS architectures remain appropriate as a low-threshold engagement layer for population-level promotion and adherence support, while clinical-grade mHealth platforms intended for diagnosis, treatment monitoring, or palliative care require bidirectional architectures with structured data models, secure patient identification, and formally specified integration interfaces with facility-based clinical information systems (85).

### 4.5 Cancer information hubs: emerging federated and interoperability architectures

Cancer information hubs and data exchange frameworks in Africa exhibit a structural distinction between centralised analytics platforms and operationally integrated data exchange architectures (86). Centralised platforms including the Global Cancer Observatory, CI5/CI5plus, the African Cancer Registry Network, and the International Cancer Control Partnership portal operate as web-based repositories characterised by unidirectional data flow: registries submit data through periodic submission processes, and processed outputs are made available for surveillance, benchmarking, and policy planning. Interoperability is achieved through standardised coding schemas (ICD-O, IARC quality criteria) rather than real-time API-based exchange. While well-suited to continental-scale cancer burden estimation, these architectures are structurally decoupled from routine clinical and registry operations submissions occur infrequently, outputs are aggregated rather than patient-level, and no feedback loop into facility-level workflows exists. They represent important reference architectures for the reporting layer but must not be conflated with the real-time, bidirectional exchange architectures required at the operational level. The DHIS2-based frameworks the IARC-DHIS2 cancer registry toolkit and the DHIS2 Tracker cervical cancer programme in Burkina Faso and Côte d’Ivoire represent a structurally superior and more operationally integrated model. By embedding cancer registry and screening functions within nationally deployed routine health information infrastructure, DHIS2 creates a unified data environment that avoids the fragmentation inherent in standalone vertical systems. The DHIS2 Tracker cervical cancer implementation is particularly instructive: its facility-laboratory notification workflow linking screening results, laboratory processing, and follow-up coordination within a single tracker architecture demonstrates the feasibility of longitudinal, individual-level cancer data management within national HIS. CanReg5, while not a hub, functions as the critical standardisation node underpinning most African PBCRs, converting diverse facility-level inputs into harmonised data models for submission to CI5 and AFCRN. The key architectural insight for developers is the need to explicitly distinguish and design for three structural layers’ operational data capture at the facility and registry level, national aggregation and analytics, and international benchmarking and reporting and to specify the interoperability interfaces between these layers rather than treating them as independent systems.

### 4.6 Genomic and precision oncology: distributed research-to-clinical architectures

Genomic and precision oncology frameworks in Africa are architecturally defined by federated network models, in which distributed nodes, research institutions, biorepositories, and bioinformatics centres operate under shared protocols and standardised computational pipelines while maintaining local data custody (87). This architecture is evident across H3Africa (75), H3ABioNet, AFBRECANE, and MADCaP, achieving interoperability through protocol harmonisation and consortium governance rather than centralised repositories or API interfaces. The federated model reflects deliberate design choices shaped by data sovereignty concerns, variable national regulatory frameworks, and the imperative to build local analytical capacity within African institutions. H3ABioNet exemplifies this approach through its distributed bioinformatics node structure, while the African Pharmacogenomics Network demonstrates how federated architecture sustains translational precision medicine goals across multiple sites without sacrificing cross-site comparability. A critical structural gap, however, exists between the research-facing federated architecture of genomic frameworks and the clinical-facing architectures of hospital OIS and registry systems: genomic data generated through H3Africa and AFBRECANE protocols does not flow into clinical decision support systems, tumour boards, or registry databases in any structured or routine manner. For developers, three architectural imperatives follow: federated network architecture remains the appropriate model for multi-country genomic frameworks (88); bioinformatics pipeline standardisation must be treated as a first-class architectural component; and future platforms must incorporate explicitly designed clinical translation interfaces structured pathways enabling genomic findings to be surfaced within clinical workflows, tumour board discussions, and cancer registry records without which genomic frameworks will remain structurally isolated from the clinical systems required to operationalise their findings.

Beyond their technical characteristics, the architectures identified in this review have important implications for cancer epidemiology and surveillance quality. Interoperable and higher-maturity registry and oncology information systems architectures improve cancer incidence estimation, data completeness, longitudinal follow-up, and survival analysis through better linkage of hospital, laboratory, and registry data sources (89). In contrast, fragmented and siloed systems contribute to incomplete patient tracking and weak outcome documentation (90). Similarly, bidirectional mHealth and tele-oncology platforms may strengthen follow-up adherence and continuity of care, improving the quality and reliability of longitudinal oncology datasets across African health systems as in developed economies (7). Technology adoption across Africa is strongly influenced by geographic and socioeconomic context (91). Urban and better-resourced settings are more likely to support advanced interoperable oncology systems, while rural and lower-resource settings often rely on mobile-first, decentralised, and low-bandwidth digital architectures. These differences substantially shape system design, scalability, and implementation across African health systems.

### 4.7 Limitations of the study

Although multiple databases were searched, some digital oncology frameworks operating in Africa may not have been captured, particularly locally implemented, non-indexed, unpublished, or institution-specific systems. The review is also subject to potential publication and selection bias, as frameworks with published documentation or international visibility were more likely to be identified, while some non-English implementations were underrepresented. In addition, architectural configurations, digital maturity levels, and AI integration categories were based on qualitative interpretation of reported system descriptions. Although predefined coding criteria and independent reviewer assessment were used, some degree of subjective judgement was unavoidable, particularly where technical details were incompletely reported. Many included studies also provided limited information regarding interoperability, governance, cybersecurity, and backend system integration. Furthermore, formal risk of bias and sensitivity analyses were not conducted because the review primarily focused on architectural mapping and qualitative synthesis rather than intervention effectiveness. Consequently, variations in methodological detail across included studies may have influenced framework classification and interpretation. Finally, this review focused primarily on architectural organisation and digital maturity rather than comparative effectiveness or clinical outcomes. While some frameworks reported improvements in workflow, screening uptake, or coordination, the available evidence lacked consistent outcome evaluation, implementation science metrics, and long-term sustainability assessment. Consequently, the findings should be interpreted as a structured architectural mapping of digital oncology systems in Africa rather than an evaluation of clinical effectiveness of the presented digital oncology frameworks.

## 5 Conclusion

This scoping review provides a systematic characterisation of digital oncology framework architectures across Africa, mapping how six framework categories (population-based cancer registries, hospital-based OIS, tele-oncology and virtual tumour board platforms, mHealth frameworks, cancer information hubs, and genomic and precision oncology systems) are structurally organised and what those architectures imply for future platform development. The findings reveal a landscape shaped by recurring structural patterns: centralised single-node registries, vendor-integrated closed-loop OIS, hub-and-spoke tele-oncology, unidirectional mHealth broadcast systems, layered analytics hubs, and federated genomic networks, each with distinct strengths, limitations, and scalability pathways.

Four architectural conclusions carry the greatest implications for platform developers, health system architects, and policymakers. First, the dominant challenge facing African digital oncology is not the absence of digital systems but their structural fragmentation: functional platforms exist across all six categories but lack the inter-system interfaces, shared data standards, and integration architectures required to constitute coherent, longitudinal, patient-centred data environments. The DHIS2 integration models and Rwanda’s national HIS-embedded cancer registry offer concrete architectural precedents for the required shift from designing isolated vertical platforms to designing interoperable components of a shared oncology infrastructure. Second, architectural maturity, not merely technological presence, determines functional value: the characteristics distinguishing Digital Maturity Level 3 from Level 2 systems; API-based interoperability, multi-site infrastructure, national HIS embedding, and structured output interfaces reflect design decisions that are neither technically complex nor cost-prohibitive, and should be treated as minimum viable requirements for new platform development rather than aspirational enhancements. Third, AI-readiness must be embedded as a structural design principle from the outset: the current absence of AI integration across all reviewed categories is, in part, an architectural legacy of systems not designed to generate the standardised, longitudinal, patient-linked data that machine learning requires. Standardised data models, persistent patient identifiers, metadata capture, and data governance frameworks must be specified at the architecture stage to create the conditions for future AI training, validation, and deployment. Fourth, and fundamentally, architecture is a determinant of health data equity. The geographic concentration of higher-maturity systems in well-resourced tertiary facilities, the exclusion of lower-resourced and rural settings from interoperable data environments, and the structural isolation of genomic frameworks from clinical and registry systems collectively constitute an architectural equity deficit; one in which the populations bearing the greatest cancer burden generate the least usable digital cancer data and derive the least benefit from digitally enabled care. Addressing this deficit requires that health data equity be treated as an explicit architectural design objective: platforms must be designed not only to function within well-resourced environments but to generate, exchange, and translate cancer intelligence equitably across the full spectrum of African health system contexts. This demands inclusive data standards that accommodate low-bandwidth and low-resource settings, governance frameworks that ensure community and patient data sovereignty, and investment models that prioritise architectural equity alongside technical performance.

The reviewed frameworks represent a substantial foundation for digital cancer control in Africa. Translating that foundation into an integrated, interoperable, AI-enabled, and equitable oncology ecosystem will require deliberate architectural investment that is explicitly aligned with both the scale of Africa’s cancer burden and the imperative that the benefits of digital oncology be distributed justly across all populations it is designed to serve.

## Data Availability

The original contributions presented in the study are included in the article/Supplementary material, further inquiries can be directed to the corresponding author.
