# Supplementary material for: Digital oncology frameworks in Africa: a scoping review of architectural patterns, digital maturity, and data equity implications
Source: Front Public Health. 2026 May 28;14:1838736. doi: 10.3389/fpubh.2026.1838736 (PMC13254031; doi:10.3389/fpubh.2026.1838736)
Supplement: Supplementary file 1 [file Data_Sheet_1.PDF]

PubMed/MEDLINE
("digital oncology"[Title/Abstract] OR "oncology information system"[Title/Abstract]
OR "cancer registry"[Title/Abstract] OR "tele-oncology"[Title/Abstract]
OR "mHealth cancer"[Title/Abstract] OR "precision oncology"[Title/Abstract])
AND
("system architecture"[Title/Abstract] OR "health information system"[Title/Abstract]
OR interoperability[Title/Abstract] OR "platform design"[Title/Abstract]
OR "data exchange"[Title/Abstract])
AND
(Africa[Title/Abstract] OR "sub-Saharan Africa"[Title/Abstract]
OR "low- and middle-income countries"[Title/Abstract])

Web of Science
TS=("digital oncology" OR "oncology information system"
OR "cancer registry" OR "tele-oncology"
OR "mHealth cancer" OR "precision oncology")
AND
TS=("system architecture" OR "health information system"
OR interoperability OR "platform design"
OR "data exchange")
AND
TS=(Africa OR "sub-Saharan Africa"
OR "low- and middle-income countries")

IEEE Xplore
("All Metadata":"digital oncology" OR "oncology information system"
OR "cancer registry" OR "tele-oncology"
OR "mHealth cancer" OR "precision oncology")
AND
("system architecture" OR "health information system"
OR interoperability OR "platform design"
OR "data exchange")
AND
(Africa OR "sub-Saharan Africa"
OR "low- and middle-income countries")

ScienceDirect
("digital oncology" OR "oncology information system"
OR "cancer registry" OR "tele-oncology"
OR "mHealth cancer" OR "precision oncology")
AND
("system architecture" OR "health information system"
OR interoperability OR "platform design"
OR "data exchange")
AND
(Africa OR "sub-Saharan Africa"
OR "low- and middle-income countries")

African Journals Online (AJOL)
("digital oncology" OR "oncology information system"
OR "cancer registry" OR "tele-oncology"
OR "mHealth cancer" OR "precision oncology")
AND
("system architecture" OR "health information system"
OR interoperability OR "platform design"
OR "data exchange")
AND
(Africa OR "sub-Saharan Africa"
OR "low- and middle-income countries")
