# Supplementary material for: Digital oncology frameworks in Africa: a scoping review of architectural patterns, digital maturity, and data equity implications
Source: Front Public Health. 2026 May 28;14:1838736. doi: 10.3389/fpubh.2026.1838736 (PMC13254031; doi:10.3389/fpubh.2026.1838736)
Supplement: Supplementary file 2 [file Data_Sheet_2.PDF]

Supplementary File 2. Full Data Extraction Sheet for Included Digital Oncology Frameworks

ID	Framework	Country/Region	Category	Architecture	Data Flow	Interoperability	Digital Maturity	AI Level	Scale	Cancer Continuum Coverage
1	South Africa National Cancer Registry	South Africa	PBCR	Centralised national registry	Mostly unidirectional	Limited	Level 3	Level 0	National	Surveillance/research
2	Kampala Cancer Registry	Uganda	PBCR	Centralised CanReg5	Unidirectional	Limited	Level 2	Level 0	Regional	Surveillance/research
3	Nairobi Cancer Registry	Kenya	PBCR	Centralised CanReg	Unidirectional	Limited	Level 2	Level 0	Urban/regional	Surveillance/research
4	Gharbiah Cancer Registry	Egypt	PBCR	Centralised regional registry	Unidirectional	Standardised registry coding	Level 3	Level 0	Regional	Surveillance/research
5	Casablanca Cancer Registry	Morocco	PBCR	Centralised registry	Unidirectional	Limited	Level 3	Level 0	Regional	Surveillance/policy
6	Harare Cancer Registry	Zimbabwe	PBCR	Centralised CanReg	Unidirectional	Limited	Level 2	Level 0	Regional	Surveillance/research
7	Blantyre Cancer Registry	Malawi	PBCR	Centralised registry	Unidirectional	Limited	Level 2	Level 0	Regional	Surveillance/research
8	Rwanda National Cancer Registry	Rwanda	PBCR	Hybrid centralised HIS-linked	Unidirectional /mixed	National HIS-linked	Level 3	Level 0	National	Surveillance/planning

**9**	Seychelles Cancer Registry	Seychelles	PBCR	Centralised national registry	Unidirectional	Limited	Level 3	Level 0	National	Surveillance
**10**	Ibadan Cancer Registry	Nigeria	PBCR	Centralised registry	Unidirectional	Standardised registry coding	Level 2	Level 0	Regional	Surveillance/research
**11**	Kumasi Cancer Registry	Ghana	PBCR	Centralised electronic registry	Unidirectional	Limited	Level 2	Level 0	Regional	Surveillance/research
**12**	Addis Ababa Cancer Registry	Ethiopia	PBCR	Centralised CanReg	Unidirectional	Limited	Level 2	Level 0	Regional	Surveillance
**13**	Bamako Cancer Registry	Mali	PBCR	Centralised registry	Unidirectional	Limited	Level 2	Level 0	Regional	Surveillance
**14**	Antananarivo Cancer Registry	Madagascar	PBCR	Centralised registry	Unidirectional	Limited	Level 2	Level 0	Regional	Surveillance/research
**15**	Butaro Cancer Centre OIS	Rwanda	Hospital OIS	Centralised OpenMRS oncology module	Bidirectional internal	Limited/OpenMRS-based	Level 2	Level 0	Referral centre	Diagnosis/treatment/ monitoring
**16**	NSIA-LUTH Varian ARIA	Nigeria	Hospital OIS	Departmental vendor-integrated	Bidirectional internal	Vendor-based	Level 3	Level 0	Tertiary centre	Radiotherapy/treatment
**17**	National Hospital Abuja MOSAIQ	Nigeria	Hospital OIS	Departmental vendor-integrated	Bidirectional internal	Vendor-based	Level 3	Level 0	Tertiary hospital	Radiotherapy/treatment
**18**	Ocean Road Cancer Institute EMR	Tanzania	Hospital OIS	Facility-based	Bidirectional internal	Limited	Level 2	Level 0	National cancer centre	Diagnosis/treatment

				centralised EMR						
**19**	Uganda Cancer Institute ARIA	Uganda	Hospital OIS	Centralised radiotherapy platform	Bidirectional internal	Vendor-based/limited external	Level 2	Level 0	National cancer institute	Diagnosis/treatment
**20**	National Cancer Institute Cairo HIS	Egypt	Hospital OIS	Integrated hospital information system	Bidirectional internal	Limited	Level 2	Level 0	Tertiary institute	Diagnosis/treatment/imaging
**21**	Groote Schuur Hospital ARIA	South Africa	Hospital OIS	Departmental vendor-integrated	Bidirectional internal	Vendor-based	Level 3	Level 0	Tertiary hospital	Radiotherapy/treatment
**22**	Equra Healthcare MOSAIQ cluster	South Africa	Hospital OIS	Multi-site centralised cluster	Bidirectional internal/networked	Vendor-based	Level 3	Level 0	Multi-site network	Radiotherapy/treatment
**23**	Kenyatta National Hospital MOSAIQ	Kenya	Hospital OIS	Departmental centralised platform	Bidirectional internal	Vendor-based/limited external	Level 3	Level 0	National referral hospital	Radiotherapy/treatment
**24**	Africa Cancer Research and Control ECHO	Multi-country Africa	Tele-oncology/VTB	Hub-and-spoke telementoring	Bidirectional communication	Platform-based	Level 2	Level 0	Continental	Diagnosis/treatment planning/capacity building
**25**	Francophone cervical/breast cancer e-learning platform	Francophone Africa	Tele-oncology/VTB	Virtual mentorship/tumour board	Bidirectional communication	Platform-based	Level 2	Level 0	Multi-country	Women’s cancers/training

**26**	IGCS Project ECHO Global Curriculum	Multi-country Africa	Tele-oncology/VTB	Hub-and-spoke ECHO model	Bidirectional communication	Platform-based	Level 2	Level 0	Multi-site	Gynecologic oncology/treatment planning
**27**	Zambia virtual gynecologic oncology tumour board	Zambia	Tele-oncology/VTB	Virtual MDT + dashboard/referral tracking	Bidirectional	Dashboard-based	Level 2–3	Level 0	National/subnational	Diagnosis/treatment planning/navigation
**28**	Butaro telepathology triage system	Rwanda	Telepathology	Hub-and-spoke digital pathology	Bidirectional consultation	Image/file-based	Level 2	Level 0	Hospital-specialist network	Diagnosis
**29**	Rwanda multidisciplinary tumour boards	Rwanda	Tele-oncology/VTB	Institutionalised MDT coordination	Bidirectional communication	Limited/platform-based	Level 2	Level 0	National/referral network	Treatment planning
**30**	Tanzania ultrasound virtual case discussion	Tanzania	Tele-oncology/virtual review	Virtual case-review training model	Bidirectional communication	Platform-based	Level 1–2	Level 0	Proof-of-concept	Diagnostic capacity building
**31**	Ghana culturally tailored SMS screening intervention	Ghana	mHealth	Centralised one-way SMS	Unidirectional	Minimal	Level 1–2	Level 0	Urban/community	Awareness/screening
**32**	Tanzania HPV-positive follow-up SMS reminders	Tanzania	mHealth	Centralised SMS scheduling	Unidirectional	Minimal	Level 2	Level 0	Programme/pilot	Follow-up screening
**33**	Tanzania educative/rem	Tanzania	mHealth	Centralised one-way SMS	Unidirectional	Minimal	Level 1–2	Level 0	Implementation study	Awareness/screening behaviour

	inder SMS framework									
34	Zambia CCPPZ mobile messaging	Zambia	mHealth	Bulk one-way SMS	Unidirectional	Minimal	Level 2	Level 0	Programme/national-scale	Awareness/screening/re-screening
35	WHO mCervicalCancer initiative	Global/Africa-applicable	mHealth	Standardised mHealth implementation architecture	Mostly unidirectional/mixed	Programme guidance	Level 2	Level 0	Multi-country framework	Prevention/screening/follow-up
36	Smartphone digital cervicography/EVA	Zambia/SSA	mHealth/imaging	Clinic-centred smartphone imaging	Mixed/optional remote review	Image/file-based	Level 1–2	Level 0	Programme/pilot	Screening/triage
37	Uganda HPV vaccination SMS/call reminders	Uganda	mHealth	Centralised SMS/voice reminders	Unidirectional	Minimal	Level 2	Level 0	Primary prevention programme	HPV vaccination/prevention
38	Tanzania mPalliative Care Link	Tanzania	mHealth	Smartphone/web app + centralised backend	Bidirectional	App/web-based	Level 2	Level 0	Palliative care programme	Symptom monitoring/palliative care
39	Uganda PCAU mHealth surveillance	Uganda	mHealth	Mobile app reporting + central analytics	Unidirectional/mixed	App-based	Level 2–3	Level 0	Multi-site/national reporting	Palliative care surveillance
40	Nigeria mobile psychoeducation intervention	Nigeria	mHealth	Mobile educational/support platform	Unidirectional/mixed	App/mobile-based	Level 1–2	Level 0	Feasibility study	Treatment support/psychoeducation

**41**	Global Cancer Observatory	Africa/global	Information hub/data exchange	Centralised web analytics platform	Export-oriented	Download/API-like access	Level 3	Level 0	Global/continental	Surveillance/policy/research
**42**	CI5 and CI5plus	Africa/global	Information hub/data exchange	Centralised benchmarking platform	Registry-to-hub reporting	Standardised metadata/coding	Level 3	Level 0	Global registry network	Surveillance/benchmarking
**43**	African Cancer Registry Network	Sub-Saharan Africa	Information hub/data exchange	Continental coordination hub	Mixed/coordination	Standards/resource-based	Level 2–3	Level 0	Regional/continental	Surveillance capacity building
**44**	GICR Sub-Saharan Africa hub	Sub-Saharan Africa	Information hub/data exchange	Hub-based technical support architecture	Mixed/coordination	Standards/tools-based	Level 2–3	Level 0	Regional/continental	Registry development/training
**45**	CanReg5	Africa-wide	Data infrastructure	Registry-level centralised platform	Mostly unidirectional	Standardised registry format	Level 2	Level 0	Multi-country tool	Surveillance/research
**46**	DHIS2–IARC cancer registry toolkit	Africa/global	Data exchange framework	National HIS-integrated registry architecture	Mixed/bidirectional potential	DHIS2-based	Level 2–3	Level 0	Scalable national/multi-country	Surveillance/planning
**47**	DHIS2 Tracker cervical cancer programme	Burkina Faso/Côte d’Ivoire	Data exchange framework	Centralised tracker linking facilities/labs	Bidirectional programme workflow	DHIS2 internal interoperability	Level 2–3	Level 0	Multi-country programme	Screening/diagnosis/follow-up
**48**	ICCP portal	Africa/global	Information hub	Centralised policy repository	Export/search-oriented	Document-based	Level 2	Level 0	Global/policy hub	Policy/planning

**49**	H3Africa	Pan-African	Genomic/precision oncology	Distributed network with shared standards	Federated/distributed	Protocol-based	Level 3	Level 0	Continental	Genomic research/biobanking
**50**	H3ABioNet	Pan-African	Genomic/precision oncology	Federated bioinformatics network	Distributed	Shared tools/QC standards	Level 3	Level 0	Continental	Genomic analytics
**51**	AFBRECANE	Nigeria	Genomic/precision oncology	Multi-site cohort + central harmonisation	Mixed	Protocolised data models	Level 2–3	Level 0	National research network	Breast cancer genomics
**52**	MADCaP	Nigeria/Ghana/Senegal/South Africa	Genomic/precision oncology	Multi-country consortium architecture	Distributed/federated	Consortium standards	Level 3	Level 0	Multi-country	Prostate cancer genomics
**53**	African Pharmacogenomics Consortium/Network	Pan-African	Genomic/precision oncology	Network-based training/protocol architecture	Distributed	Protocol/training-driven	Level 2–3	Level 0	Continental	Pharmacogenomics/treatment optimization
